# The Italian Version of the International Trauma Questionnaire: Symptom and Network Structure of Post-Traumatic Stress Disorder and Complex Post-Traumatic Stress Disorder in a Sample of Late Adolescents Exposed to a Natural Disaster

**DOI:** 10.3389/fpsyt.2022.859877

**Published:** 2022-05-25

**Authors:** Rodolfo Rossi, Valentina Socci, Francesca Pacitti, Claudia Carmassi, Alessandro Rossi, Giorgio Di Lorenzo, Philip Hyland

**Affiliations:** ^1^Department of Systems Medicine, Tor Vergata University of Rome, Rome, Italy; ^2^Department of Biotechnological and Applied Clinical Sciences, University of L’Aquila, L’Aquila, Italy; ^3^Department of Clinical and Experimental Medicine, University of Pisa, Pisa, Italy; ^4^IRCCS Fondazione Santa Lucia, Rome, Italy; ^5^Department of Psychology, Maynooth University, Maynooth, Ireland

**Keywords:** PTSD, ICD-11 cPTSD, International Trauma Questionnaire, factor structure, network analysis

## Abstract

The 11th revision of the International Classification of Diseases has endorsed substantial changes in Post-Traumatic Stress Disorder (PTSD) and has introduced Complex PTSD (cPTSD). The objective of this study was to assess the symptom and network structure of PTSD and cPTSD using the International Trauma Questionnaire- Italian version (ITQ) and the prevalence of PTSD and cPTSD in a community sample of late adolescents enriched with exposure to a destructive earthquake. A 1,010 high school students participated to the study. Confirmatory Factor Analysis supports that a six first-order correlated factors was the best fitting model of ICD-11 PTSD/cPTSD. The network analysis supports a clear separation between core PTSD symptoms and disturbances in self-organization (DSO) symptoms, avoidance, and negative self-concept were the most central items. The prevalence of PTSD and cPTSD was 9.11 and 4.06%, respectively. Female participants reported higher rates of both PTSD and cPTSD. This is the first study to report on ICD-11 PTSD and cPTSD rates on an Italian adolescence community sample. Consistent with other community samples, we found higher rates of PTSD compared to cPTSD. The results confirmed the factorial validity of the ITQ. The network structure highlights the importance of negative self-concept in cPTSD and avoidance in PTSD.

## Introduction

Exposure to traumatic experiences (TE) is common in the general population with 70% of people globally reporting at least one potential TE in their lifetime, and 30% reporting four or more TEs (1). The prevalence of different types of TE varies across regions of the world, and across other sociodemographic variables. In developed countries, the most frequent TEs are the sudden death of a loved one, technological accidents (in particular, motor vehicle accidents), and crime-related TE such as being robbed (1). Exposure to TE is associated with a wide range of adverse mental health outcomes, and it is a criterion for a diagnosis of Post-Traumatic Stress Disorder (PTSD). Interpersonal TEs, particularly those that are repeated and/or occur early in life are especially harmful TEs and are associated with a plethora of serious adverse mental health and functional outcomes.

The 11th revision of the World Health Organization’s (WHO) International Classification of Diseases (ICD-11) included substantial changes to the “Disorders Specifically Associated with Stress” section, including a revised definition of PTSD and the introduction of Complex PTSD (cPTSD; (2)). In the ICD-11, PTSD is focused on a small set of core trauma symptoms grouped into three clusters (re-experiencing in the here and now, avoidance of traumatic reminders, and a sense of threat) (3). cPTSD includes the three symptom clusters of PTSD, plus three symptom clusters of Affective Dysregulation, Negative Self-Concept, and Disturbances in Relationships. Collectively, these three clusters are termed Disturbance in Self-Organization (DSO; (4)). DSO symptoms were selected to capture the pervasive disturbances that commonly arise from enduring interpersonal TEs from which escape or avoidance is difficult or impossible (5). In this respect, the Psychodynamic Diagnostic Manual (PMD-2; (6)) conceptualizes Complex PTSD as a developmental trauma disorder, emphasizing how traumatic events between birth and adulthood might compromise the optimal development of the individual’s identity, self-worth, personality, emotional regulation and self-regulation. While the type of TE is not used to make a differential diagnosis, PTSD and cPTSD typically differ in the types of TE that precipitate their development. TD more often associated with interpersonal traumas and early life traumas, whereas PTSD is more often associated with non-interpersonal and later life traumas (7, 8). One key feature of a TE that could inform on the pathogenetic potential is the presence of the intention to harm. Interpersonal TEs such as abuse, violence, neglect, or aggression are, by definition, deliberate acts aiming to harm an individual. On the other hand, TEs such as natural disasters or accidents are events that occur either without any input from other person or any deliberate motive to cause of harm. An interesting feature of TEs that include an intention to cause harm is that they tend to be inter-related and are therefore likely to co-occur over an extended period of time (9).

The International Trauma Questionnaire (ITQ) (10) is a self-report measure developed to capture all elements of the ICD-11 diagnoses of PTSD and cPTSD. The psychometric properties of the ITQ have been extensively investigated in community (10–12) and clinical (13, 14) samples. Despite the widespread use and support for the psychometric properties of the ITQ (for a review see Redican et al. (15)), most of this empirical support comes from the original English version of the ITQ. An explicit goal of the revisions to the ICD-11 was to maximize the global applicability of these diagnoses. Thus, it is essential that research be conducted to assess the psychometric properties of the translations of the ITQ.

This study has two main objectives. The first aim is to assess the factor and symptom structure model of the Italian version of the ITQ using a factor and network analysis. In recent years a number of studies have addressed the network structure of DSM-IV- and DSM-5-defined PTSD (16). Evidence regarding ICD-11 PTSD and cPTSD is more limited; however, converging evidence supports a clear separation between the core PTSD and the DSO symptoms (17), and negative self-concept items as central nodes in cPTSD (18, 19). Results regarding centrality in PTSD is more mixed, with hyperarousal (20) and re-experiencing (21) being reported more frequently as central nodes. Our aim is to extend these findings on a non-clinical sample of late adolescents. Our first hypothesis is that a clear factorial separation between core PTSD symptoms and DSO symptoms will be found in our sample, consistent with previous works.

The second purpose is to assess the prevalence of PTSD and cPTSD in a sample of Italian late adolescents exposed 10 years earlier to a destructive earthquake together with any gender differences. This population is characterized by collective exposure to an unintentional traumatic event in the form of a natural disaster during a critical developmental period. In this respect, we sought to extend upon current knowledge about the type of traumatic events that are associated with meeting the diagnostic criteria for PTSD and cPTSD by assessing the association between intentional and unintentional TEs with PTSD and cPTSD. In particular, the secondary hypothesis that intentional TEs would be more strongly associated with meeting criteria for cPTSD compared to unintentional TEs, which include the exposure to a natural disaster. Our expected results are that intentional TEs would exert a more relevant impact on PTSD and DSO symptoms, compared to unintentional TE.

## Methods

### Participants and Procedure

The participants in the present study were enrolled in the first time point of the Dual Trauma study at the University of L’Aquila. Dual trauma is a longitudinal study started in 2019 that focused on a target population comprising all of the late adolescents and young adults attending the last year of high school in the province of L’Aquila, central Italy, during the academic year 2019–2020. The estimated target population size is 2000. This population is of particular interest as virtually all of the population from L’Aquila and its surroundings, estimated half of the total target population, were directly exposed to the 2009 earthquake when they were between 7 and 10 years old. Most of the remaining target population coming from the surroundings of Avezzano and Sulmona were not directly exposed to this event. Inclusion criteria were attending the last year of high school and being > 18 years old at the time of the enrollment. Enrollment was limited to students > 18 who were chosen in order to not introduce any selection bias deriving from parental consensus. Exclusion criteria included being on supported teaching as a proxy of mild to severe cognitive impairment.

A clustered sampling was conducted based on geographical area (L’Aquila, Avezzano and Sulmona), school and class. In the area, 12 out of 14 high schools were identified and invited to participate; two schools did not provide consent to participate. In the remaining ten schools, 50 to 100% of the last-year classes were allowed to participate by the headmasters. Individual written consent was provided by the participants. Questionnaires were collected in paper and pencil form by the University staff.

The local ethics committee at the University of L’Aquila provided approval to the study (research number 49, 26/09/2019). This study adheres to the declaration of Helsinki.

Recruitment and data collection took place between November 2019 and January 2020. A total of 1010 adolescents participated in the study, 506 (50.15%) males, mean age 18.7 (sd = 0.65). Geographical area was distributed as follows: 418 (41.39%) from L’Aquila surroundings, 306 (30.30%) from Avezzano surroundings and 286 (28.32%) from Sulmona surroundings. 694 (68.71%) participants reported being directly exposed to the 2009 central Italy earthquake.

Of the 1,010 initial sample, eighteen participants had complete missing data on the ITQ and were thus excluded. The final analysis was performed on 992 subjects.

### Measures

#### The International Trauma Exposure Measure (ITEM)

The ITEM (22) is a 21-items checklist capturing traumatic life events, and their associated features, in a manner consistent with the ICD-11 description of a TE. The ITEM measures exposure to TEs across three developmental periods (childhood, adolescence, adulthood). The ITEM is freely available at https://www.traumameasuresglobal.com to the research and clinical communities and may be used without permission. For the purpose of this study, the following modifications were introduced: for each TE, the respondent was asked to rate whether the TE had occurred during childhood, adolescence, or in the last 6 months. Each TE was classified as intentional or unintentional by two independent psychiatrists with expertise in trauma-related research that were blind to all the study objectives and variables. The results were checked and discussed with the original authors of the ITEM. During the classification process, three TE were excluded because deemed not pertinent as intentional nor unintentional TE. Full ITEM questions, their lifetime endorsement by participants, and their classification as intentional or unintentional are reported in Table 1.

**TABLE 1:** Mean scores of ITQ items.

Factor	Variable	0 = Not at all	1 = A little	2 = Moderately	3 = Quite a lot	4 = Very much	Total mean	Std.
***PTSD***								
*Re-experiencing*	Re1 – Dreams	463 (47.1%)	266 (27.0%)	138 (14.0%)	79 (8.0%)	38 (3.9%)	0.99	1.15
	Re2 – Nightmares	338 (34.3%)	248 (25.2%)	176 (17.9%)	152 (15.4%)	71 (7.2%)	1.40	1.28
*Avoidance*	Av1 – Avoidance – internal	372 (37.8%)	233 (23.7%)	186 (18.9%)	118 (12.0%)	74 (7.5%)	1.34	1.29
	Av2 – Avoidance – external	427 (43.4%)	205 (20.8%)	157 (15.9%)	119 (12.1%)	77 (7.8%)	1.28	1.33
*Hyperarousal*	Hyp1 – Hyperarousal	463 (47.1%)	250 (25.4%)	127 (12.9%)	94 (9.6%)	50 (5.1%)	1.05	1.21
	Hyp2 – Easily startled	504 (51.3%)	209 (21.3%)	139 (14.2%)	80 (8.1%)	50 (5.1%)	1.00	1.21
***DSO***								
*Emotional Dysregulation*	Dys1 – Difficult calm down	156 (15.8%)	284 (28.7%)	263 (26.6%)	201 (20.3%)	85 (8.6%)	1.83	1.18
	Dys2 – Numbing	519 (52.5%)	216 (21.8%)	126 (12.7%)	82 (8.3%)	46 (4.7%)	0.97	1.20
*Negative Self Concept*	Nsc1 – Feel like a failure	723 (73.1%)	132 (13.3%)	66 (6.7%)	38 (3.8%)	30 (3.0%)	0.54	1.01
	Nsc2 – Worthlessness	729 (73.7%)	139 (14.1%)	58 (5.9%)	39 (3.9%)	24 (2.4%)	0.51	0.98
*Relational problems*	Rel1 – Feel cut from others	678 (68.6%)	190 (19.2%)	56 (5.7%)	46 (4.7%)	18 (1.8%)	0.55	0.95
	Rel2 – Difficult stay close to others	577 (58.3%)	238 (24.1%)	104 (10.5%)	53 (5.4%)	17 (1.7%)	0.72	1.00

#### The Italian Version of the International Trauma Questionnaire

The ITQ (10) is a self-report questionnaire measuring all aspects of the ICD-11 diagnostic criteria for PTSD and cPTSD. The ITQ first asks a participant to identify their most distressing traumatic event and how long ago this event occurred. Participants are then instructed to answer all questions in relation to that event. The ITQ includes six items to measure each of the PTSD symptoms across the clusters of Re-experiencing in the Here and Now, Avoidance of internal and external reminders, and Sense of Current Threat (Hyperarousal). Participants report how bothered they have been by each symptom over the past month. Additionally, participants indicated how much these symptoms have interfered with their ability to function in life in the past month across three items. These are six items measuring each of the DSO symptoms across the clusters of Affective Dysregulation, Negative Self-Concept, and Disturbances in Relationships (Relational problems). Participants answer these questions in terms of how they typically feel, think about themselves, and relate to others. There are also three items used to measure the extent to which these symptoms impair functioning in life over the last month. All items are based on a five-point Likert scale that ranges from 0 (Not at all) to 4 (Extremely).

A diagnosis of PTSD requires that a person was trauma exposed, the endorsement (defined as a score ≥ 2 on the Likert scale) of at least one of two symptoms in each one of the three symptoms cluster plus endorsement (defined as a score ≥ 2) of at least one indicator of functional impairment. A diagnosis of cPTSD requires the endorsement of one of two symptoms from each of the three PTSD symptoms clusters and one of two symptoms from each of the three Disturbances in Self-Organization (DSO) clusters. The functional impairment must be identified where at least one indicator of functional impairment is endorsed related to the PTSD symptoms, and one indicator of functional impairment is endorsed related to the DSO symptoms. An individual can receive either a diagnosis of PTSD or cPTSD, not both. In our sample, reliability was α = 0.88 for the PTSD subscale and α = 0.88 for the DSO subscale.

The translation process followed standard back-translation procedures, with two psychiatrists providing a draft English-to-Italian translation, a third psychiatrist produced a joint version of the two drafts, and a native English speaker provided a back-translated version that was approved by the authors. The English and Italian versions of the ITQ are freely downloadable at https://www.traumameasuresglobal.com/itq.

## Statistical Analysis

The weighted least squares (WLS) Confirmatory Factor Analysis (CFA) was conducted to evaluate the fit of four different models already presented in the literature. WLS provides accurate parameter estimates, standard error and test statistics for ordinal indicators. Based on previous findings (10, 11, 23–29), we specified four different models of PTSD and cPTSD factor structure. Model 1 is a one-factor model where all of the ITQ items loaded onto a single cPTSD factor. Model 2 is a correlated six-factor (i.e., Re-experiencing in the Here and Now, Avoidance, Sense of Current Threat, Affective Dysregulation, Negative Self-Concept, and Disturbances in Relationships) model. Model 3 is a second-order model with six first-order factors and one second-order factor of cPTSD. Model 4 is a two factor second-order model with a second-order PTSD factor and a second-order DSO factor. Model fit for the CFA was evaluated using: a non-significant chi-square result indicates good model fit; Comparative Fit Index (CFI) and Tucker Lewis Index (TLI) values ≥ 0.90 and ≥ 0.95 indicate adequate and excellent fit, respectively (30); and Root Mean Square Error of Approximation (RMSEA) values ≤ 0.08 and ≤ 0.06 indicate adequate and excellent fit. CFA analysis was performed using STATA^®^ v.16 (StataCorp (31)).

A network analysis on the 12 ITQ symptoms items was conducted using an Extended Bayesian Information Criterion Graphical Least Absolute Shrinkage and Selection Operator (EBICglasso) estimator. EBICglasso (32) is a partial correlation regularized estimation method that estimates partial correlations among all variables in the network, which use penalized maximum likelihood estimation to shrink parameters to zero, potentially removing them from the network. EBICglasso tuning parameter was set to 0.5. Centrality measures estimated included Betweenness (i.e., the number of shortest paths that pass through the node of interest), Closeness (i.e., the inverse of the sum of all shortest paths from the node of interest to all other nodes), and Strength (i.e., the sum of the absolute input weights of that node).

Finally, prevalence estimates for PTSD and cPTSD were estimated, together with gender differences. Association with traumatic experiences and a screened diagnosis of PTSD or cPTSD was assessed using multinomial logistic regression models, with ITQ categorical results (no diagnosis, PTSD and cPTSD) modeled as dependent variable, and intentional and unintentional TEs experienced during childhood, adolescence or during the last 6 months were jointly modeled as independent variables in order to correct their effects for each other. Afterward, analyses were adjusted by gender, parental education, and nationality.

All of the statistical analyses were conducted using Stata 16^®^, except for the network analysis that was conducted using JASP^®^.

## Results

### Sample

A total of 1,010 subjects participated to the study, 999 of which provided complete data – 498 (49.85%) female and 501 (50.15%) males. Mean age was 18.7 (sd = 0.63). 418 (41.39%), 306 (30.30%), and 286 (28.32%) were, respectively, from the L’Aquila, Avezzano and Sulmona areas.

### Confirmatory Factor Analysis

Confirmatory Factor Analysis goodness-of-fit statistics for the four models are presented in Table 1. Model 1 (*χ^2^* = 1903.43, RMSEA = 0.188, CFI = 0.619, TLI = 0.535, SRMR = 0.109, CD = 0.868, BIC = 32985.043) and 3 (*χ^2^* = 517.447, RMSEA = 0.101, CFI = 0.903, TLI = 0.867, SRMR = 0.076, CD = 0.942, BIC = 31640.318) showed poor fit indices, while Model 2 (*χ^2^* = 175.18, RMSEA = 0.06, CFI = 0.972, TLI = 0.953, SRMR = 0.036, CD = 0.999, BIC = 31359.942) and 4 (*χ^2^* = 256.186, RMSEA = 0.068, CFI = 0.957, TLI = 0.94, SRMR = 0.055, CD = 1, BIC = 31385.933) showed good fit indices, although the χ^2^ statistic was significant due to the large sample size (33). Models 2 and 4 had very similar fit indices, with a DBIC = 25.99. Model 2 should be preferred to Model 4 as it had lower BIC and ΔBIC > 10, which is considered as a threshold for a significant difference.

### Network Analysis

**FIGURE 1:**
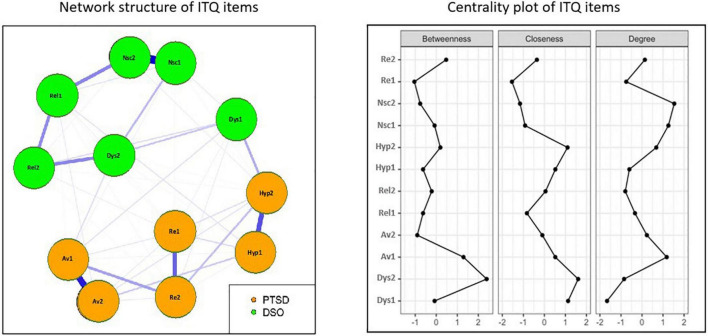
Network structure and centrality measures of ITQ items.

Network analysis and centrality plot are reported in Figure 1. From the visual inspection of the network structure, it appears that PTSD and DSO symptoms are organized in two separate clusters, and that the two nodes of each subdomain are strongly connected. The network was moderately sparse, with 46/60 non-zero edges and a sparsity of 0.31. Among the core PTSD symptoms, item Av1- “Avoidance of internal reminders” had the highest betweenness and strength (betweenness = 1.294, closeness = 0.517, strength = 1.175). Centrality measures for the remaining core PTSD items were: Av2- “Avoidance – external” (betweenness = −0.904, closeness = −0.1, strength = 0.23); Reexperiencing, Re1- “Dreams” (betweenness = −1.042, closeness = −1.544, strength = −0.751) and Re2- “Nightmares” (betweenness = 0.469, closeness = −0.359, strength = 0.134); Hyp1- “Hyperarousal” (betweenness = −0.63, closeness = 0.519, strength = −0.602) and Hyp2- “Easily startled” (betweenness = 0.195, closeness = 1.1, strength = 0.677). Among the DSO symptoms, Dys2- “Numbing” had the highest betweenness and closeness (betweenness = 2.393, closeness = 1.604, strength = 0.853), and Nsc2 – “Worthlessness” had the highest strength (betweenness = −0.767, closeness = −1.161, strength = 1.532). For the remaining DSO items, centrality measures were: Dys1- “Difficult calm down” (betweenness = −0.08, closeness = 1.125, strength = −1.663); Nsc1- “Feel like a failure” (betweenness = −0.08, closeness = −0.919, strength = 1.255); Rel1- “Feel cut from others” (betweenness = −0.63, closeness = −0.834, strength = −0.335); Rel2- “Difficulty stay close to others” (betweenness = −0.218, closeness = 0.052, strength = −0.797). Items with the highest expected influence were both “negative self-concept” Nsc1- “feel like a failure” and Nsc2- “worthlessness” items, Av1- Avoidance of internal reminders and hyperarousal item Hyp2-easily startled. In the whole network, the most influential nodes were Dys2 – “Numbing” with the highest betweenness and closeness, and the two Nsc items.

### Prevalence of Post-Traumatic Stress Disorder and Complex Post-Traumatic Stress Disorder and Association With Traumatic Experiences (TEs)

Detailed rates of traumatic experiences and psychopathology separated by gender are reported in Table 2. Overall, 914 (90.5%) of participants reported at least one lifetime traumatic experience, the most frequent being exposure to a natural disaster (597; 60.49%), and the least represented frequent being exposed to war or combat (2; 0.2%). No participant reported sexual abuse from a parent, while 32 participants (3.21%) reported a sexual assault by someone other than a parent. Females were more exposed to intentional TE compared to male participants (*χ^2^* = 19.847, *p* < 0.001), while no gender differences were reported for unintentional TE. In particular, female participants reported higher rates of sexual assault and harassment, bullying, humiliation and neglect. Male participants reported higher rates of being threatened with a weapon and being physically assaulted by a non-parent.

**TABLE 2:** Lifetime traumatic experiences, psychopathology and gender differences in the sample.

Variable		Male		Female		Total		Statistics
				
		*N/Mean*	*%/sd*	*N/Mean*	*%/sd*	*N/Mean*	*%/sd*	*χ^2^ or t*
**Lifetime victimization**								
1. Life-threatening illness	Unint.	10	2.00%	16	3.22%	26	2.61%	1.471
2. Someone close died in an awful manner	Unint.	91	18.20%	154	30.99%	245	24.57%	21.983***
3. Someone close had a life-threatening illness or accident	Unint.	217	43.49%	247	49.70%	464	46.59%	3.860*
4. Threatened with a weapon	Int.	37	7.39%	17	3.41%	54	5.41%	7.704**
5. Physically assaulted by a parent or guardian	Int.	37	7.41%	41	8.23%	78	7.82%	0.231
6. Physically assaulted by non-parent or guardian	Int.	84	16.80%	45	9.05%	129	12.94%	13.274***
7. Sexually assaulted by a parent or guardian	Int.	0	0%	0	0%	0	0%	–
8. Sexually assaulted by non-parent or guardian	Int.	10	2.00%	22	4.42%	32	3.21%	4.698*
9. Sexually harassed	Int.	14	2.80%	66	13.25%	80	8.02%	36.972***
10. War or combat	Int.	2	0.40%	0	0.00%	2	0.20%	1.996
11. Held captive and/or tortured	Int.	2	0.40%	1	0.20%	3	0.30%	0.330
12. Caused extreme suffering or death to another person.	Excl.	7	1.40%	3	0.60%	10	1.00%	1.608
13. Witnessed another person experiencing suffering or death	Excl.	55	11.02%	80	16.06%	135	13.54%	5.412*
14. Technological accident	Unint.	89	17.84%	58	11.67%	147	14.76%	7.524**
15. Natural disaster	Unint.	288	57.95%	309	63.06%	597	60.49%	2.699
16. Man-made disaster	Unint.	30	6.02%	19	3.89%	49	4.96%	2.391
17. Stalked	Int.	35	7.06%	50	10.06%	85	8.56%	2.861
18. Bullied (online or offline)	Int.	60	12.07%	88	17.71%	148	14.89%	6.224*
19. Humiliated, put down, or insulted by another person	Int.	142	28.63%	194	39.03%	336	33.84%	12.005***
20. Made to feel unloved, unwelcome, or worthless	Int.	162	32.60%	249	50.20%	411	41.39%	31.720***
21. Neglected, ignored, rejected, or isolated	Int.	141	28.54%	199	40.28%	340	34.41%	15.085***
Lifetime Unintentional TE		404	80.64%	421	84.54%	825	82.58%	2.640
Lifetime Intentional TE		269	53.69%	336	67.47%	605	60.56%	19.847***
Lifetime TE		444	88.62%	461	92.57%	905	90.59%	4.5657*
**Prevalence**								
PTSD		28	5.63%	63	12.96%	91	9.26%	15.712***
cPTSD		10	2.01%	30	6.17%	40	4.07%	10.897***

*p < 0.05; **p < 0.01; ***p < 0.001.

International Trauma Questionnaire prevalence rates are reported in Table 1, while detailed descriptive statistics are reported in Table 2. According to the diagnostic algorithm, the prevalence of PTSD and cPTSD was 9.11% [7.54, 11.25] and 4.06% [2.98, 5.56], respectively. There was a significant gender effect, with female participants having higher rates of both PTSD and cPTSD (respectively, *χ^2^* = 15.712, *p* < 0.001 and *χ^2^* = 10.897, *p* < 0.001).

Logistic regression analyses are reported in Table 3. Unintentional TEs, independently of the age of occurrence, were not associated with a PTSD or a cPTSD diagnosis. Intentional TE, irrespectively of the age of occurrence, were associated with both PTSD and cPTSD. Association between childhood and recent intentional TE and PTSD did not hold after adjustment.

**TABLE 3:** Association between PTSD, cPTSD and traumatic experiences (multinomial logistic regression).

	PTSD^a^	cPTSD^a^	PTSD^b^	cPTSD^b^
	*OR [95% CI]*	*OR [95% CI]*	*OR [95% CI]*	*OR [95% CI]*
**Intentional TE**				
*Childhood*	1.75* [1.10, 2.76]	2.49* [1.20, 5.15]	1.57 [0.98, 2.51]	2.63* [1.23, 5.59]
*Adolescence*	2.43*** [1.51, 3.89]	3.53*** [1.67, 7.49]	2.36*** [1.45, 3.84]	3.48** [1.61, 7.52]
*6 months*	2.25** [1.36, 3.72]	4.46*** [2.25, 8.85]	2.19** [1.31, 3.66]	4.36*** [2.15, 8.83]
**Unintentional TE**				
*Childhood*	1.34 [0.79, 2.28]	0.65 [0.32, 1.31]	1.19 [0.69, 2.05]	0.77 [0.36, 1.63]
*Adolescence*	0.95 [0.60, 1.51]	1.34 [0.69, 2.61]	1.01 [0.62, 1.64]	1.38 [0.68, 2.78]
*6 months*	1.06 [0.59, 1.90]	1.17 [0.53, 2.60]	1.17 [0.65, 2.12]	1.41 [0.62, 3.19]

*p < 0.05; **p < 0.01; ***p < 0.001. TE, traumatic experiences; OR, odds ratio; CI, confidence interval. ^a^Unadjusted logistic regression; ^b^adjusted by gender, non-Italian nationality, parental education.

## Discussion

The present study is the first to report on prevalence rates of ICD-11 PTSD and cPTSD within an Italian late adolescent sample primarily exposed to trauma in the form of a natural disaster, and their association with different types of TEs. Additionally, this study adds to the existing literature on the factorial and network structure of the ICD-11 PTSD and cPTSD on an Italian late adolescent/young adult population.

The ITQ is a brief, highly reproducible instrument that allows to jointly screen for PTSD and cPTSD according to the ICD-11 criteria. In our sample, PTSD was nearly twice as frequent as cPTSD, and both diagnoses were more frequent among female participants. This finding is in line with previous reports from a nationally representative Israeli sample of a higher prevalence of PTSD compared to cPTSD (11). Other studies found opposite results, with cPTSD being more frequent than PTSD (3, 7, 10, 24, 34). However, these studies, except for the Chinese validation study and the United Kingdom general population study (10), focused on clinical populations rather than a general population, suggesting that cPTSD may be a more common issues among help-seeking populations compared to the general population (27). Concerning the Chinese validation study (34), PTSD and cPTSD prevalence were estimated only on the general population subsample that reported at least one adverse childhood experience (ACE), excluding those subjects that may have been exposed to unintentional or non-interpersonal TEs that may not qualify as an ACE that may endorse PTSD but not DSO criteria. The United Kingdom study focused on both a clinical and a community sample. However, in this case the community sample was an adult nationally representative one, while the present study reports on a late adolescence sample.

In the present study, regression analyses support that both intentional and unintentional TEs are associated with a diagnosis of cPTSD, although intentional TEs show a stronger association with cPTSD. Conversely, we failed to demonstrate an association between unintentional TE and a diagnosis of PTSD. These results could be explained by the fact that a TE screener such as the ITEM may effectively screen for the presence of any TE, but it may fail to separate those TE that had a relevant psychological impact from those that didn’t. In our sample, which is enriched with youngsters that experienced a massively destructive earthquake during childhood, unintentional TE had a lifetime prevalence of 82% and a 6-months prevalence of 15.35%, compared to a PTSD prevalence of 9.11%. It follows that the vast majority of those experiencing unintentional TEs do not develop PTSD or, in other words, the vast majority of unintentional TE is not inherently associated with a relevant psychological burden sufficient for a diagnosis of PTSD.

As opposite, intentional TEs were strongly associated with both PTSD and cPTSD.

The present CFA results support a latent structure representative of six first-order correlated factors. The second best-fitting model was a two-factor second-order model. According to a recent systematic review (15), these two models are those most often identified as the best fitting ones. The correlated six first-order factor model was supported in five community sample studies (26, 34–37), in which also the two-factor second-order model showed good fit. Two more studies found that the two models had equivalent fit (25, 38). Evidence from the literature seem to converge on the six first-order factor model as the best fitting model in community samples, while the two-factor second-order model being the best model in clinical samples (15). Furthermore, prevalence rates of PTSD and cPTSD are substantially lower in community samples, which may explain why the separation between PTSD and cPTSD is not so clear-cut in these samples.

Finally, using a network analysis approach, we replicated the network structure of ICD-11 PTSD and cPTSD previously presented in other studies with the ITQ (17, 19). Given the contrasting findings on the factor structure of ICD-11 PTSD and cPTSD, the not clear-cut separation of the two disorders, and their shared risk factors, a network approach gains a central importance in clarifying the mutual relationships among stress-related symptoms. A network approach is based on the idea that mental disorders are constituted by causal networks of functionally interconnected symptoms, as opposite to a “latent variable” representing the putative disorder that generates downstream symptoms (39). The network approach to PTSD and cPTSD is particularly useful as it allows to think of the effects of causal factors (i.e., traumatic events) as spreading throughout a network of interacting and mutually influencing symptoms. In the network structure presented in this study, the pairs of symptoms forming a factor according to the ICD-11 were strongly associated each other. Core PTSD symptoms and DSO form two broader clusters, reflecting the ICD-11 proposed criteria that separates core PTSD and DSO, consistently with previous literature evidence (17). Avoidance, numbing and both negative self-concept (Nsc) were the most central items in the network. The centrality of Nsc items in ICD-11 cPTSD and DSM-5 PTSD has been found also in other network studies (19, 40, 41). Nsc can be thought both as negative moderators of the effect of a TE, and as a cognitive maintaining factor of the disorder. Furthermore, Nsc are among the strongest negative predictors of treatment outcomes (42). Concerning core PTSD symptoms, having internal avoidance as the most central item seems counterintuitive, as avoidance is generally considered a coping mechanism to anxiety. Its central position in this network may highlight its role as a maintaining factor.

This study has several limitations. First, one third of this sample consists of late adolescents that were exposed to a natural disaster during childhood, making the generalizability of these findings limited. Second, this study relies on self-report measures that could be affected by different biases. In particular, despite a TEs screener was available, too little is known about the putative TEs subsiding the self-report measures of post-traumatic symptoms, in terms of subjective emotional impact and relative importance of the TEs. It is difficult to imagine how to overcome these issues connected with self-report measures. However, the ITEM checklist is a useful tool to separate childhood, adolescence and recent TEs and to classify them into different types (i.e., intentional/unintentional).

## Conclusion

The present study reports a prevalence rate Post-Traumatic Stress Disorder (PTSD) and Complex PTSD (cPTSD) of 9.11 and 4.06%, respectively, in a sample of Italian adolescents exposed 10 years earlier to a destructive earthquake. The factor and network structure of the Italian version of the International Trauma Questionnaire (ITQ) confirmed the factorial validity of the questionnaire, highlighting the importance of avoidance in PTSD and negative self-concept in cPTSD.

## Data Availability Statement

The raw data supporting the conclusions of this article will be made available by the authors, without undue reservation.

## Ethics Statement

The studies involving human participants were reviewed and approved by the Internal Review Board of the University of L’Aquila. The patients/participants provided their written informed consent to participate in this study.

## Author Contributions

RR: conceptualization, methodology, software, validation, formal analysis, investigation, data curation, and writing – original draft. VS: data curation, writing, editing, and review. FP, CC, AR, and PH: writing, editing, and review. GD: conceptualization and supervision. All authors contributed to the article and approved the submitted version.

## Conflict of Interest

The authors declare that the research was conducted in the absence of any commercial or financial relationships that could be construed as a potential conflict of interest.

## Publisher’s Note

All claims expressed in this article are solely those of the authors and do not necessarily represent those of their affiliated organizations, or those of the publisher, the editors and the reviewers. Any product that may be evaluated in this article, or claim that may be made by its manufacturer, is not guaranteed or endorsed by the publisher.
